# Molecular mechanisms of cell death by parthanatos: More questions than answers

**DOI:** 10.1590/1678-4685-GMB-2023-0357

**Published:** 2024-08-30

**Authors:** Rafael Dias de Moura, Priscilla Doria de Mattos, Penélope Ferreira Valente, Nícolas Carlos Hoch

**Affiliations:** 1 Universidade de São Paulo, Instituto de Química, Departamento de Bioquímica, São Paulo, SP, Brasil.

**Keywords:** Cell death, parthanatos, PARP1, PARG, AIF

## Abstract

Regulated cell death by a non-apoptotic pathway known as parthanatos is increasingly recognised as a central player in pathological processes, including ischaemic tissue damage and neurodegenerative diseases. Parthanatos is activated under conditions that induce high levels of DNA damage, leading to hyperactivation of the DNA damage sensor PARP1. While this strict dependence on PARP1 activation is a defining feature of parthanatos that distinguishes it from other forms of cell death, the molecular events downstream of PARP1 activation remain poorly understood. In this mini-review, we highlight a number of important questions that remain to be answered about this enigmatic form of cell death.

## Introduction

ADP-ribosylation (ADPr) is a covalent modification of biological macromolecules catalysed by members of the ADP-ribosyltransferase family, which transfer ADP-ribose moieties from NAD+ (nicotinamide adenine dinucleotide) to target proteins or nucleic acids (Hoch and Polo 2019; Luscher et al., 2021; Suskiewicz et al., 2023). ADP-ribosyltransferases can be subdivided based on the nature of the ADPr modification they catalyse, which can be in the form of single ADP-ribose units, termed mono(ADP-ribose) (MAR) or as long and sometimes branched chains of poly(ADP-ribose) (PAR). The main human PAR-catalysing enzyme is poly(ADP-ribose) polymerase 1 (PARP1), which is a highly abundant nuclear protein that consists of three DNA-binding zinc finger domains (ZnF1, ZnF2, and ZnF3), a central BRCA1 C-terminal (BRCT) domain, a DNA-binding WGR (tryptophan, glycine, arginine) domain and a bipartite C-terminal catalytic domain composed of an auto-inhibitory helical subdomain (HD) and an ADP-ribosyl transferase (ART) subdomain. PARP1 plays central roles in the cellular response to DNA damage, due to the high affinity and specificity of its DNA-binding domains for DNA strand breaks, which lead to rapid and robust activation of PARP1 catalytic activity in response to a variety of DNA lesions (Pandey and Black 2021; Pascal 2023). Once activated, PARP1 modifies itself and a growing list of chromatin-associated proteins, including core histones, promoting the recruitment of PAR-binding DNA repair proteins to the lesion site and accelerating DNA repair (Ray Chaudhuri and Nussenzweig 2017; Hendriks et al., 2021; Caldecott, 2022). Interestingly, there is extensive literature on roles of PARP1 in many other cellular processes, such as chromatin remodelling, gene regulation and inflammation (Hottiger, 2015; Fehr et al., 2020; Kim et al., 2020), but whether PARP1 is also responding to some form of DNA damage under these conditions or if PARP1 can be catalytically activated in the absence of a DNA strand break is currently unclear.

In addition to its canonical role in accelerating DNA repair and, therefore, promoting cell survival in response to DNA lesions, PARP1 can become hyperactivated in response to high levels of acute DNA damage, triggering a regulated form of cell death termed parthanatos (Yu et al., 2002; Fatokun et al., 2014). In this setting, genetic PARP1 deletion or pharmacological PARP1 inhibition are strongly cytoprotective, and this strict PARP1 dependency is the defining feature of parthanatos that distinguishes it from other forms of cell death, such as apoptosis or necrosis (Fatokun *et al.,* 2014). 

Parthanatos can be triggered by several exogenous or endogenous sources that generate a high load of PARP1-activating DNA breaks, such as the alkylating agents MNNG (N-methyl-N’-nitro-N-nitrosoguanidine) or MMS (methyl methanesulfonate), or a variety of treatments that induce high bursts of reactive oxygen or nitrogen species, such as hydrogen peroxide (H_2_O_2_) and other oxidants. In neuronal cells, this can be achieved by overstimulation of glutamate receptors via NMDA (N-methyl-D-aspartate) or glutamate, in a process also known as glutamate excitotoxicity (Mandir et al., 2000). Several disease models that rely on DNA damage-induced tissue dysfunction, such as steptozotocin-induced diabetes and MPTP-induced Parkinsonism also rely on parthanatos for tissue demise (Yamamoto et al., 1981; Wang et al., 2003). Ischemia-reperfusion is another well-documented process that induces a burst of oxidative DNA damage, leading to PARP1-mediated cell death (Eliasson et al., 1997; Dawson and Dawson, 2018) and more recently, it has become evident that PARP1 hyperactivation and parthanatos play a pathological role in neurodegenerative disorders as well, including Parkinson’s disease and Alzheimer’s disease (Hoch et al., 2017; Kam et al., 2018; Park *et al.,* 2020, 2022). 

**Figure 1 -:**
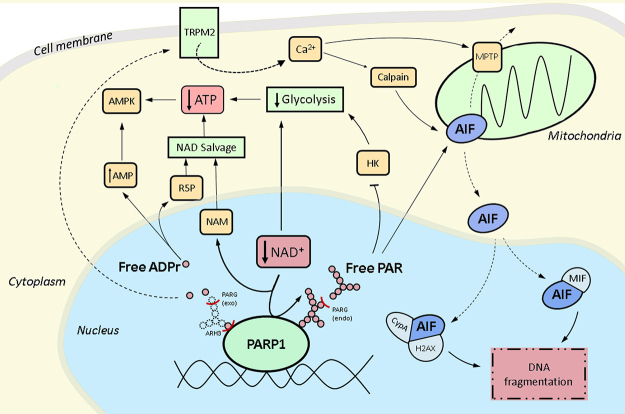
Potential pathways of cell death mediated by PARP1 hyperactivation.

The variety of pathophysiological situations that lead to PARP1 hyperactivation and the likely clinical benefit of PARP inhibitors to manage these disorders have been extensively reviewed (Fatokun et al., 2014; Berger et al., 2018; Liu et al., 2022 a , b), and will only be briefly mentioned here. Likewise, other genetic or pharmacological interventions that affect the magnitude of spontaneous or induced PARP1 hyperactivation will not be discussed (Andrabi et al., 2011; Kang et al., 2011; Yang et al., 2022). Instead, this short review will focus on mechanistic considerations of the downstream events that follow excessive PARP1 catalytic activity and how they contribute to cellular demise (Figure 1). At the end of each section, we will provide a list of open questions that have not been addressed so far or that are currently unclear from the literature.

PARP1 hyperactivation depletes NAD+ levels and generates protein-linked PAR chains. The hydrolases PARG and ARH3 can produce free PAR chains and/or free ADP-ribose monomers. Both NAD+ depletion and free PAR chains are thought to inhibit glycolysis, which, together with NAD+ salvage, could lead to ATP depletion. Free ADP-ribose monomers can be cleaved by Nudix hydrolases, contributing to NAD+ salvage and the generation of AMP. The ensuing changes in the AMP/ATP ratio can activate AMP-activated kinase (AMPK). Free ADPribose monomers can promote TRPM2 channel opening, increasing cytoplasmic calcium levels. This could activate calpain and/or induce opening of the mitochondrial permeability transition pore (MPTP). Free PAR chains, calpain activation or the MPTP could promote AIF release from the mitochondria, leading to AIF translocation to the nucleus. AIF/MIF or AIF/cyclophilinA/H2AX complexes could then mediate DNA fragmentation. Please refer to the text for more details. NAM: nicotinamide; R5P: ribose 5-phosphate; HK: hexokinase. Note: NAD+ salvage could, in principle, occur both in the nucleus or cytoplasm, but was drawn as shown for simplicity.

## NAD+ depletion and inhibition of glycolysis

Since NAD+ is consumed in the process of ADP-ribosylation, donating the ADP-ribose moiety for target modification, and because PARP1 is a highly abundant and processive enzyme, PARP1 hyperactivation results in a rapid and profound depletion of cellular NAD+ pools (Berger 1985). Interestingly, this is accompanied by a depletion of cellular ATP, indicating that parthanatos could be a result of cellular energetic collapse (Berger 1985; Ha and Snyder 1999). 

One possible explanation for PARP1-dependent ATP depletion is the unavailability of NAD+ to act as an electron acceptor for core metabolic pathways such as glycolysis and the tricarboxylic acid (TCA) cycle, with the accompanying reduction of the available NADH for oxidative phosphorylation. Early studies using astrocyte cultures indicated NAD+ depletion as the primary mediator of parthanatos, as parthanatos-associated events could be induced by other NAD+-depleting treatments and prevented by supplementation with exogenous NAD+ (Alano et al., 2004, 2010; Ying et al., 2005). Treatment with pyruvate or α-ketoglutarate, which can support TCA cycle activity but bypass glycolysis, was also sufficient to prevent cell death, indicating a glycolytic defect. As the authors pointed out, however, this scenario could be limited to situations in which glucose is the only substrate for energy metabolism in the culture medium used (artificial cerebrospinal fluid in this case), while conventional media often contain pyruvate and other carbon sources. Similarly, Zong et al. (2004) observed that cells that rely more heavily on glycolysis are more susceptible to cell death by parthanatos, which can be reversed by supplementation with pyruvate. Further evidence indicating a central role for NAD+ levels in parthanatos, is the observation that treatment with the NAD+ precursors nicotinamide riboside (NR) or nicotinamide mononucleotide (NMN) can prevent PARP1-dependent cell death in some settings (Nishida et al., 2022; Santofimia-Castaño et al., 2022). In this context, it is worth mentioning that cellular NAD+ pools are compartmentalized, and that nuclear and cytoplasmic NAD+ are in rapid equilibrium, whereas mitochondrial NAD+ pools are maintained separately (Cambronne et al., 2016; Covarrubias et al., 2021). This would indicate that nuclear PARP1 hyperactivation should impact the nuclear/cytoplasmic NAD+ pool more rapidly, which would be consistent with a larger impact of parthanatos-induced NAD+ depletion on cytoplasmic glycolysis than on mitochondrial TCA cycle. However, NAD+ can be transported across the mitochondrial membrane via the recently identified SLC25A51 transporter (Girardi et al., 2020; Kory et al., 2020; Luongo et al., 2020), and there is evidence for a mitochondrial pool of PARP1 (Szczesny et al., 2014; Herrmann et al., 2021; Lee et al., 2022), suggesting that PARP1-dependent depletion of mitochondrial NAD+ may also play a role in parthanatos execution. 

Other studies in cortical neurons and glioblastoma-derived cell lines have suggested that NAD+ depletion itself is not sufficient to cause ATP depletion, glycolytic defects or cell death, only being responsible for defects in mitochondrial respiration (Andrabi *et al.,* 2014; Fouquerel et al., 2014). In these studies, profound NAD+ depletion in the absence of PARP1 hyperactivation was insufficient to induce parthanatos and supplementation with nicotinamide riboside (NR), an NAD+ precursor, did not prevent glycolytic dysfunction (Andrabi *et al.,* 2014; Fouquerel *et al.,* 2014). In this scenario, PARP1 activity is thought to directly inhibit the first step of glycolysis, via the release of PAR polymers from target proteins by PAR-degrading enzymes (see below), which then bind to hexokinase-1 and inhibit its catalytic activity (Andrabi *et al.,* 2014; Fouquerel *et al.,* 2014). Although at odds with the NAD+-centric model that emerged from the above studies, supplying cells with pyruvate was again sufficient to overcome PARP1-mediated metabolic dysfunction, consistent with glycolysis being a core target of parthanatos (Andrabi *et al.,* 2014) (Figure 1). As hexokinase also generates substrates for the pentose phosphate pathway (PPP), this mechanism is also consistent with the recently described depletion of reduced glutathione (GSH) and NADPH during parthanatos, which are both products of the PPP (Hossain et al., 2024). 

Another model to explain the proposed uncoupling between ATP and NAD+ depletion derives from the observation that AMP, which may be generated at high levels during PAR chain degradation (see below), can inhibit the mitochondrial adenine nucleotide translocator (ANT) (Formentini et al., 2009; Buonvicino et al., 2013). This would lead to an impaired translocation of ADP into the mitochondria, inhibiting mitochondrial ATP synthesis due to the low availability of ADP for oxidative phosphorylation. 

Interestingly, it has been suggested that ATP depletion is responsible for diverting cells from apoptosis to parthanatos, with PARP1 hyperactivation thus acting as a “switch” between these forms of cell death (Ha and Snyder 1999). In agreement with this, recent findings indicate that lower (but still cytotoxic) levels of DNA damage induce an intermediate level of NAD+ consumption by PARP1 that can be matched by the NAD+ salvage pathway, leading to a transient NAD+ and ATP depletion that allows cell death to proceed by apoptosis, whereas higher DNA damage loads cause a more prolonged NAD+ and ATP depletion that precludes apoptosis (Nishida et al., 2022). Conversely, PARP1 hyperactivation is also actively prevented during the apoptotic cascade via caspase-dependent cleavage of PARP1 between the DNA binding domains and the catalytic domain, which is thought to ensure that the cell can meet the energy requirements of apoptosis (D’Amours et al., 2001).

Open questions:

- What factor(s) determine(s) whether NAD supplementation does or does not prevent parthanatos induction?
- Is the inhibition of glycolysis necessary and/or sufficient for cell death by parthanatos?
- How do free PAR chains inhibit hexokinase activity?
- Are NAD+ and ATP depletion mechanistically connected?
- Is there more extensive crosstalk between apoptosis and parthanatos, or is this limited to parthanatic NAD+/ATP depletion preventing apoptosis and apoptotic PARP1 cleavage preventing parthanatos?

### PAR hydrolysis

The human genome encodes several hydrolases responsible for the reversal of ADP-ribosylation: those belonging to the macrodomain family - PARG, TARG1, MacroD1 and MacroD2; and those in the ADP-ribose-acceptor hydrolase family - ARH1 and ARH3 (O’Sullivan et al., 2019; Rack et al., 2020). Among these, PARG and ARH3 are crucial for the hydrolysis of PARP1-generated PAR chains, with PARG contributing the bulk of PAR hydrolysis activity, cleaving the O-glycosidic bond between ADP-ribose units, both in linear chains as well as at branching points (Rack *et al.,* 2021). While ARH3 can also contribute to PAR hydrolysis, its main role is the release of the final serine-bound mono-ADP-ribose (Abplanalp et al., 2017; Fontana et al., 2017), serine being the main target residue for PARP1 in response to DNA damage (Palazzo et al., 2018). Similar to PARP1, PARG is a highly active enzyme, making poly-ADP-ribosylation a very transient modification that is produced and degraded within minutes of an insult (Hanzlikova et al., 2018). Interestingly, the reversal of DNA damage-induced mono-ADP-ribosylation, which can be generated either as a remnant of PARG activity or directly by PARP1, seems to be much slower, indicating a longer-lasting, and therefore different, cellular response (Longarini et al., 2023). 

There is extensive but conflicting evidence as to the role of PARG in parthanatos, with several studies suggesting that PARG can either prevent or promote PARP1-dependent cell death. In favour of an inhibitory role, PARG overexpression reduced MNNG-induced cell death in mouse neuronal cultures (Andrabi et al., 2006) and reduced NMDA-induced AIF release from mitochondria (Yu et al., 2006), an important step in most parthanatos models, which is covered in more detail below. Similarly, knockdown of PARG increased cell death in mouse neurons (Andrabi *et al.,* 2006) and PARG deletion in trophoblast stem cells increased AIF release after UV irradiation (Zhou et al., 2011). PARG +/- mice had larger infarct volumes after brain ischaemia-reperfusion injury, while mice overexpressing PARG had smaller infarct volumes (Andrabi *et al.,* 2006). In contrast, other studies suggest that PARG is necessary for, or at least contributes to, the process of parthanatos. PARG inhibition protected mice against brain ischaemia (Lu et al., 2003), and PARG silencing rendered cells more resistant to treatment with H_2_O_2_ but not MNNG (Blenn et al., 2006). In the aforementioned studies proposing the hexokinase inhibition model, there is also conflicting evidence regarding the role of PARG. In (Fouquerel et al., 2014), PARG knockdown rescued the PARP1-dependent glycolytic defect and ATP depletion, while in (Andrabi *et al.,* 2014) a similar rescue of glycolysis was observed after PARG overexpression. Table 1 shows a compilation of results regarding the contribution of PARG to PARP1-dependent cell death and associated effects in different models, highlighting the considerable heterogeneity currently in the literature. 

**Table 1 -:** Contribution of PARG activity, AIF translocation and TRPM2 channel gating to cell death by parthanatos. Only studies in which the induced cell death was shown to rely on PARP1 activity have been included.

Model	Insult	Effect of PARG on cell death	AIF translocation	TRPM2 contribution to cell death	Reference
Mouse embryos	MNNG, menadione	Protective (knockout increased cell death)	-	-	Koh et al., 2004
MEFs	H_2_O_2_	Protective (knockdown increased cell death	-	-	Blenn et al., 2006
Cortical neurons	NMDA	Protective (overexpression reduced cell death	-	-	Andrabi et al., 2006
Mice	middle cerebral artery occlusion (MCAO)	Protective (heterozygous KO increased tissue damage; overexpression reduced tissue damage)	-	-	Andrabi et al., 2006
Cortical neurons	MNNG	Protective (overexpression prevented glycolytic defect)	-	-	Andrabi *et al.,* 2014
MEFs	MNNG	No effect	-	-	Blenn et al., 2006
HK-2 cells	TGHQ	No effect	-	-	Munoz et al., 2017
Mice and rats	splanchnic artery occlusion (SAO) shock	Detrimental (knockout and inhibition protected tissues)	-	-	Cuzzocrea et al., 2005
Glioblastoma cells	MNNG	Detrimental (knockdown prevented ATP depletion and glycolytic defect)	-	-	Fouquerel et al., 2014
Glioblastoma cells	MMS	Protective (knockdown increased cell death)	No	-	Tang et al., 2010
Pancreatic cancer cells	ZZW-115 (NUPR1 inhibitor)	Protective (inhibition increased cell death)	No	-	Santofimia-Castaño et al., 2022
Trophoblast stem cells	UV	Protective (knockout increased cell death)	Yes	-	Zhou et al., 2011
MEFs	H_2_O_2_	Detrimental (knockdown reduced cell death)	Yes	-	Mashimo et al., 2013
Rat fibroblasts	MNNG	-	Yes	-	Yu et al., 2002
Neurons	NMDA	-	Yes	-	Yu et al., 2006
MEFs	H_2_O_2_	-	Yes	-	Kolthur-Seetharam et al., 2006
MEFs	MNNG	-	Yes	-	Wang et al., 2009
Cortical neurons	NMDA	-	Yes	-	Wang et al., 2011
Glioma cells	DPT	-	Yes	-	Ma et al., 2016
SH-SY5Y cells	MNNG	-	Yes	-	Zhong et al., 2018
HK-2 cells	TGHQ	-	No	-	Zhang et al., 2014
Retinal cells	H_2_O_2_	-	No	-	Jang et al., 2017
Mouse bone-marrow derived macrophages	H_2_O_2_	-	No	-	Regdon et al., 2019
HEK293 expressing recombinant TRPM2	H_2_O_2_	-	-	Increased	Fonfria et al., 2004
Rat striatal neurons	H_2_O_2_ and amyloid β-peptide(1-42)	-	-	Increased	Fonfria et al., 2005
Rat cardiomyocytes	H_2_O_2_	-	-	Increased (apoptosis markers also present)	Yang et al., 2006
Mice	MCAO	-	-	Increased infarct volumes (also androgen signalling-dependent)	Shimizu et al., 2013
RIN-5F (rat pancreatic β-cells)	H_2_O_2_	-	-	Increased	Ishii et al., 2014
Mouse hippocampal neurons	H_2_O_2_	-	-	Increased (death is also partly Zn^2+^ dependent)	Li et al., 2017
SH-SY5Y overexpressing TRPM2	H_2_O_2_	-	-	Increased	An et al., 2019

These competing roles of PARG can, at least in theory, be ascribed to two separate functions that both depend on PARG catalytic activity, but have opposing effects on parthanatos execution. One possibility is centered around the formation of free PAR chains, which are thought to inhibit hexokinase (above) and release AIF from mitochondria (below) (Figure 1). PARG activity could be required for the formation of these free PAR chains via its endoglycohydrolase activity and therefore promote parthanatos, but high PARG activity may also degrade these free PAR chains after their formation, and therefore reduce cell death by parthanatos (Mashimo et al., 2013). In this context, it is worth mentioning that, while PARG acts both as an exo- and endoglycohydrolase, its exoglycohydrolase activity is thought to be predominant (Barkauskaite et al., 2015), indicating that PAR hydrolysis generates mostly ADP-ribose monomers, not free PAR chains. Moreover, high levels of nuclear PARG catalytic activity imply that any free PAR polymers generated in the nucleus must be protected from PARG activity in order to reach the cytosol at any significant amounts. A further complication in the interpretation of the contribution of PARG to parthanatos is that long-term PARG deletion can affect the activation of PARP1, since PARP1 auto-modification inhibits its DNA binding, such that the accumulation of spontaneously auto-modified PARP1 in PARG KO cells can reduce the population of PARP1 molecules that can engage in DNA damage-induced PARylation (Gogola et al., 2018). 

ARH3, on the other hand, is thought to play a protective role in parthanatos, which is ascribed to its PAR-degrading activity, which would reduce the accumulation of free PAR polymers and prevent parthanatos induction (Mashimo et al., 2013) (Figure 1). In agreement with this model, ARH3-deficient mice are more sensitive to ischaemia-reperfusion injury and ARH3-deficient human patients present neurodegenerative disorders and their fibroblasts are more sensitive to H_2_O_2_-induced parthanatos (Danhauser et al., 2018; Ghosh et al., 2018). However, an alternative explanation for neurodegeneration in these patients could be that failure to remove mono-ADP-ribosylation from core histones leads to epigenetic changes that culminate in transcription deregulation (Hanzlikova et al., 2020), which would be independent of parthanatos. 

Open questions:

- Do PAR hydrolases, and PARG in particular, promote or inhibit parthanatos execution?
- How are free PAR chains generated at sufficiently high amounts, protected from hydrolytic enzymes and then transported out of the nucleus?

## ADP-ribose monomers and Ca2+ release

While the nicotinamide moiety of NAD+ released during PAR synthesis is predominantly recycled back to NAD+ by the NAD+ salvage pathway (Covarrubias et al., 2021), the ADP-ribose moiety transferred onto target proteins and subsequently released by PAR/MAR hydrolases generates free ADP-ribose monomers (Figure 1). This free ADP-ribose can bind to the calcium channel TRPM2, which contains two ADP-ribose binding sites that regulate channel opening (Perraud et al., 2001; Huang et al., 2018; Szollosi 2021). In several cell types, increases in intracellular Ca^2+^ were observed upon oxidative stress, were accompanied by the accumulation of free ADP-ribose and relied on PARP1 activation and TRPM2 gating (Fonfria et al., 2004; Perraud *et al.,* 2005; Yang et al., 2006). Although in some systems there is evidence for ADP-ribose-independent, but oxidative stress-dependent TRPM2 opening (Wehage et al., 2002), the ADPr-dependent activation requires PARG activity (Blenn et al., 2011), arguing in favour of a PARP1/PARG-dependent route of ADP-ribose generation. Consistent with this, induction of parthanatos using MNNG can also lead to calcium influx (Chiu et al., 2011). Interestingly, in a model of renal ischaemia/reperfusion injury, cell death can be prevented both by PARP1 inhibition or Ca^2+^ chelation, suggesting an important role of Ca^2+^ influx for parthanatos execution (Zhang et al., 2014). In another study, Ca^2+^ chelation only suppressed cell death upon H_2_O_2_ treatment, but not upon MNNG treatment, indicating that this effect could be specific to particular insults (Bentle et al., 2006). A further complication in the interpretation of this data is that Ca^2+^ may affect PARP1 activation by a poorly understood mechanism (Zhang *et al.,* 2014). While TRPM2 is a cell-membrane resident channel and therefore can only cause Ca^2+^ influxes from the extracellular space, Ca^2+^ release from the endoplasmic reticulum may also contribute to parthanatos (Munoz et al., 2017; Zhong et al., 2018). Interestingly, unlike TRPM2 gating, this effect was independent of PARG, suggesting a different mechanism of channel opening (Zhang *et al.,* 2014; Munoz *et al.,* 2017).

Open questions:

- Is ADP-ribose-induced TRPM2 gating necessary and/or sufficient for parthanatos execution?
- Are there TRPM2-dependent and TRPM2-independent modes of parthanatos?
- What are the downstream molecular effects of TRPM2-mediated increases in intracellular Ca^2+^?

## ADP-ribose degradation into AMP

**Figure 2 -:**
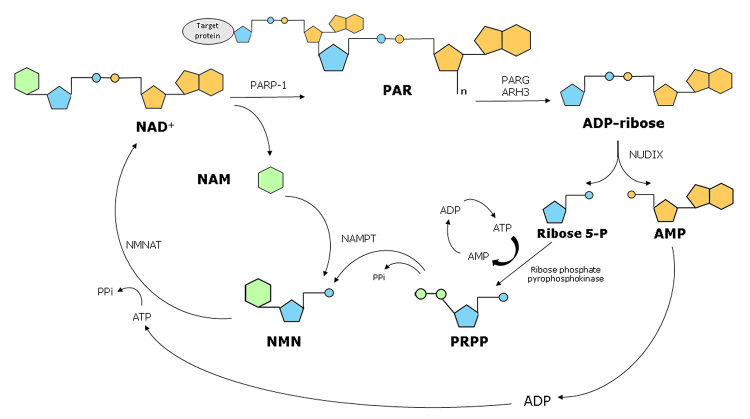
A full cycle of NAD+ salvage costs four high-energy phosphate groups per NAD+ molecule consumed by PARP1.

Free ADP-ribose can also be further degraded into AMP and ribose-5-phosphate by phosphodiesterases of the Nudix superfamily (Carreras-Puigvert et al., 2017) (Figure 1). These enzymes target the phosphodiester bond in ADP-ribose and can degrade either free ADPr or leave a phosphoribose modification on previously ADP-ribosylated proteins, although the detection of protein phosphoribose modification is currently limited to *in vitro* reactions (Daniels et al., 2015; Palazzo et al., 2015; O’Sullivan et al., 2019). The resultant ribose 5-phosphate could have many metabolic fates, including the formation of phosphoribosyl pyrophosphate (PRPP), which is required for the NAD+ salvage pathway (Figure 2). Interestingly, the complete cycle of NAD+ salvage, from conversion of NAD+ to an ADP-ribose unit by PARP1 back to a full NAD+ molecule using the same carbon backbones, has an energetic cost of four high-energy phosphate groups per ADP-ribose unit attached to a protein (Figure 2). Given that NAD(H) concentrations are roughly in the 0.3 mM range (Yang et al., 2007), while ATP concentrations are only around 10x higher, in the 3-4 mM range (Greiner and Glonek, 2021), the full consumption of cellular NAD+ by PARP1 and its subsequent salvage couId make a substantial contribution to ATP depletion during parthanatos. While the relative contributions of this Nudix-dependent salvage pathway as opposed to glycolysis inhibition (see above) to energetic collapse during parthanatos is unclear, the accumulation of ADP and particularly AMP may be an important signal in cell death after PARP1 hyperactivation (Formentini et al., 2009). Illustrating this, MNNG treatment of HEK-293 cells led to activation of the AMP-activated kinase (AMPK) attributed to increased AMP/ATP ratios, which then inhibited the mTORC1 signaling pathway, involved in the regulation of cell death/survival and energy metabolism (Ethier et al., 2012) (Figure 1). While this would suggest the induction of an autophagic response, as observed in some parthanatos models (Zhou et al., 2013; Jiang et al., 2018), whether AMPK activation and autophagy contribute to cell death execution by parthanatos or are protective mechanisms is currently unclear. 

PARP1 activity generates PAR and releases nicotinamide (NAM). PAR is cleaved into ADP-ribose monomer by PARG and ARH3, which is further cleaved by Nudix hydrolases into ribose 5-phosphate (R5P) and AMP. While the AMP is re-phosphorylated to ATP, at the cost of two high-energy phosphate groups, the R5P is converted to phosphoribosyl pyrophosphate (PRPP), at the cost of two further high-energy phosphates. PRPP is conjugated to NAM to form nicotinamide mononucleotide (NMN) and NMN is conjugated with ATP to generate NAD+. NAMPT: nicotinamide phosphoribosyltransferase; NMNAT: Nicotinamide mononucleotide adenylyltransferase.

Open questions:

- Are Nudix hydrolases required for parthanatos execution?
- What is the relative contribution of AMP generated from ADP-ribose hydrolysis, as opposed to ATP depletion from glycolysis inhibition (above), for the AMPK activation/autophagy observed in parthanatos?
- How do AMPK activation and autophagy affect cell death by parthanatos?

## AIF translocation and DNA fragmentation

Apoptosis-Inducing Factor (AIF) is a mitochondrial flavoprotein that plays a role in the assembly of the respiratory chain complexes, but is also involved in cell death mechanisms (Susin et al., 1999; Vahsen et al., 2004). It is normally located in the inner mitochondrial membrane, facing the inter-membrane space (Otera et al., 2005), but can also be found loosely associated with the outer mitochondrial membrane (Yu et al., 2009). In response to PARP1 hyperactivation, AIF is often observed to translocate from the mitochondria to the nucleus (Yu *et al.,* 2002), but some models of PARP1-dependent cell death do not lead to observable AIF translocation, indicating that there may be AIF-dependent and AIF-independent forms of parthanatos (Table 1). For example, retinal cells and macrophages do not seem to exhibit AIF translocation after PARP1-dependent cell death induction (Jang et al., 2017; Regdon et al., 2019). Interestingly, AIF translocation to the nucleus is also observed in response to some apoptotic stimuli (Daugas et al., 2000), which was recently suggested to also rely on PARP1 activation (Mashimo et al., 2021). 

In the context of parthanatos, AIF is thought to be released from mitochondria via direct interaction with free PAR polymers (above) (Yu et al., 2006; Wang et al., 2011), but the molecular details of this process are currently unclear. Alternatively, AIF release may proceed via its proteolysis, which has been observed in some situations to rely on calpain I, which is a Ca^2+^ -dependent protease, and therefore could in principle respond to TRPM2-dependent Ca2+ influxes or endoplasmic reticulum calcium release (Polster et al., 2005; Norberg et al., 2008; Vosler et al., 2009; Sun et al., 2018). However, there is rather strong evidence against a central role of calpain cleavage on AIF release, at least in some parthanatos models (Wang *et al.,* 2009). Another possible contributor to AIF release from mitochondria is the mitochondrial permeability transition pore, which is an ill-defined molecular entity that allows the non-selective diffusion of small molecules through the mitochondrial inner membrane, which can lead to mitochondrial swelling and rupture, and is associated to several cell death mechanisms (Yu *et al.,* 2006; Bernardi et al., 2023) (Figure 1). 

AIF translocation to the nucleus is associated with large scale DNA fragmentation, culminating in cell death. Two mechanisms for AIF-induced DNA cleavage have been proposed (Figure 1). One model suggests that cytoplasmic AIF interacts with macrophage migration inhibitory factor (MIF), leading to the nuclear translocation of MIF, which was identified to have a nuclease activity (Wang et al., 2016). In agreement with this model, a specific inhibitor of MIF nuclease activity was recently shown to protect cells from parthanatos in a mouse model of parkinsonism (Park et al., 2022). The second model is based on the recent identification of a nuclease activity in AIF itself, which is proposed to degrade DNA in a complex formed between AIF, cyclophilin A and histone H2AX (Artus et al., 2010; Novo et al., 2022).

Open questions:

- What is the precise sequence of molecular events that promotes AIF release from mitochondria?
- How does translocation of AIF promote DNA fragmentation and what protein(s) catalyse(s) DNA cleavage?
- What factor(s) define(s) AIF-dependent and AIF-independent parthanatos and what process leads to DNA fragmentation in AIF-independent parthanatos?
- What are the differences and similarities between apoptotic and parthanatic AIF translocation?

## Conclusions

In the late 1970s, Goodwin and colleagues first showed that DNA damage can induce the depletion of NAD and ATP levels, and that PARP1 activity is central to this effect (Goodwin *et al.,* 1978). Almost 50 years of research since then have led to the identification of a range of different stimuli that induce PARP1 hyperactivation and a variety of pathological situations in which PARP1 activation seems to contribute to cell death and tissue damage. However, several questions and inconsistencies still remain regarding the sequence of molecular events that drive cell death by parthanatos. While a number of key mechanisms have already been described, it remains unclear which events are necessary and sufficient for parthanatos execution and how each of these steps connects to the next one in the cascade. Complicating matters even further, there seem to be clear differences in how parthanatos proceeds in different cell types and at different metabolic states. With this review, we aim to highlight the urgent need for studies that determine the contribution of several steps along the cascade in single, well-defined model systems. Only by comparing all of these steps between different models in which NAD+ supplementation, PARG activity, AIF translocation or TRPM2 gating play differential roles, can we hope to shed light on whether there are multiple pathways of parthanatos or if a single pathway integrates all of these events. Although technically difficult and inherently multidisciplinary, this will be critical to better understand not only how this pathway operates, but also how other cell death mechanisms, such as apoptosis, are interconnected to parthanatos. A better definition of these mechanisms will be central to clarify the contribution of PARP1-dependent cell death to human pathology, particularly in a variety of neurodegenerative disorders, such as Parkinson’s and Alzheimer’s disease, which are of rising concern in an aging human population.
